# Diaqua­(2,6-dihy­droxy­benzoato-κ^2^ *O* ^1^ *,O* ^1′^)bis­(2,6-dihy­droxy­benzoato-κ*O* ^1^)bis­(1,10-phenanthroline-κ^2^ *N*,*N*′)lanthanum(III)–1,10-phenanthroline (1/1)

**DOI:** 10.1107/S1600536810047318

**Published:** 2010-11-20

**Authors:** Yaling Cai, Weiqin Dong, Hongxiao Jin

**Affiliations:** a College of Materials Science and Engineering, China Jiliang University, Hangzhou 310018, People’s Republic of China

## Abstract

In the title compound, [La(C_7_H_5_O_4_)_3_(C_12_H_8_N_2_)_3_(H_2_O)_2_]·C_12_H_8_N_2_, the La^III^ atom is coordinated by four N atoms from two chelating 1,10-phenanthroline (phen) ligands, four O atoms from three 2,6-dihy­droxy­benzoate (DHB) anions (one monodentate, the other bidentate) and two water O atoms, completing a distorted LaN_4_O_6_ bicapped square-anti­prismatic geometry. Within the mononuclear complex mol­ecule, intra­molecular π–π stacking inter­actions are observed, the first between a coordinated phen mol­ecule and a DHB ligand [centroid–centroid distance = 3.7291 (16) Å], and the second between a coordinated phen mol­ecule and an uncoordinated phen ligand [centroid–centroid distance = 3.933 (2) Å]. Inter­molecular π–π stacking is observed between adjacent complexes [inter­planar distance = 3.461 (3) Å]. Intra- and inter­molecular O—H⋯O hydrogen bonds are observed in the DHB ligands and between a water mol­ecule and DHB ligands, respectively. O—H⋯N hydrogen bonds are also observed in the DHB ligands and between uncoordinated phen mol­ecules and aqua ligands.

## Related literature

For background to the chemistry of lanthanide-based metal-organic frameworks, see: dos Santos *et al.* (2008 ▶). For related structures, see: Nie *et al.* (2010 ▶); Li *et al.* (2005 ▶); Zheng & Jin (2003 ▶).
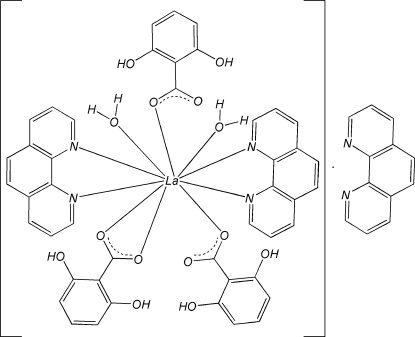


## Experimental

### 

#### Crystal data

[La(C_7_H_5_O_4_)_3_(C_12_H_8_N_2_)_3_(H_2_O)_2_]·C_12_H_8_N_2_
*M* *_r_* = 1174.88
Monoclinic, 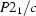

*a* = 13.9735 (3) Å
*b* = 19.6751 (3) Å
*c* = 18.6337 (3) Å
β = 98.059 (2)°
*V* = 5072.37 (16) Å^3^
*Z* = 4
Mo *K*α radiation
μ = 0.92 mm^−1^
*T* = 291 K
0.50 × 0.46 × 0.42 mm

#### Data collection

Oxford Diffraction Gemini S Ultra diffractometer
Absorption correction: multi-scan [*ABSPACK* in *CrysAlis PRO RED* (Oxford Diffraction, 2006 ▶)] *T*_min_ = 0.657, *T*_max_ = 0.699
32940 measured reflections
10312 independent reflections
7901 reflections with *I* > 2σ(*I*)
*R*_int_ = 0.022

#### Refinement

*R*[*F* ^2^ > 2σ(*F* ^2^)] = 0.031
*wR*(*F* ^2^) = 0.086
*S* = 1.05
10312 reflections
721 parameters
25 restraints
H atoms treated by a mixture of independent and constrained refinement
Δρ_max_ = 0.95 e Å^−3^
Δρ_min_ = −0.49 e Å^−3^

### 

Data collection: *CrysAlis PRO CCD* (Oxford Diffraction, 2006 ▶); cell refinement: *CrysAlis PRO CCD*; data reduction: *CrysAlis PRO RED* (Oxford Diffraction, 2006 ▶); program(s) used to solve structure: *SHELXS97* (Sheldrick, 2008 ▶); program(s) used to refine structure: *SHELXL97* (Sheldrick, 2008 ▶); molecular graphics: *DIAMOND* (Brandenburg & Berndt, 1999 ▶); software used to prepare material for publication: *SHELXL97*.

## Supplementary Material

Crystal structure: contains datablocks I, global. DOI: 10.1107/S1600536810047318/vm2058sup1.cif

Structure factors: contains datablocks I. DOI: 10.1107/S1600536810047318/vm2058Isup2.hkl

Additional supplementary materials:  crystallographic information; 3D view; checkCIF report

## Figures and Tables

**Table 1:** Hydrogen-bond geometry (Å, °)

*D*—H⋯*A*	*D*—H	H⋯*A*	*D*⋯*A*	*D*—H⋯*A*
O11—H11⋯O9	0.82	1.83	2.557 (3)	147
O12—H12⋯O10	0.82	1.80	2.520 (3)	146
O3—H3⋯O1	0.82	1.84	2.576 (3)	149
O7—H7⋯O5	0.82	1.82	2.545 (3)	147
O8—H8⋯O6	0.82	1.85	2.575 (4)	147
O4—H4⋯O2	0.82	1.78	2.512 (3)	147
O13—H13*A*⋯O10	0.87 (1)	1.92 (2)	2.704 (3)	150 (4)
O13—H13*B*⋯O2	0.86 (4)	2.11 (3)	2.762 (3)	132 (4)
O14—H14*A*⋯N6	0.86 (2)	1.96 (2)	2.793 (4)	162 (4)
O14—H14*B*⋯N5	0.83 (2)	2.26 (4)	2.824 (4)	125 (4)

**Fig. 1.**
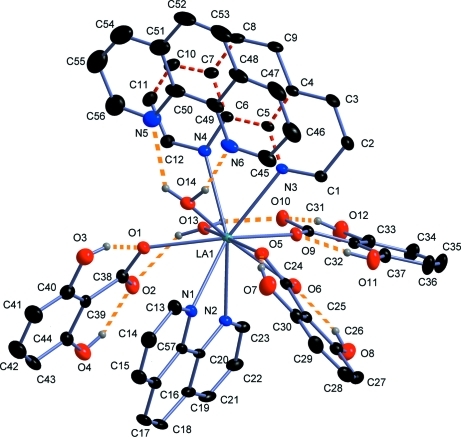
The structure, showing the atom-labelling scheme of the asymmetric unit. Displacement ellipsoids are drawn at the 15% probablility level and H atoms are shown as small spheres of arbitraty radii. Some H atoms are omitted for clarity. Bonds in overlapping phenantholine rings are shown as red dashed lines.

**Fig. 2.**
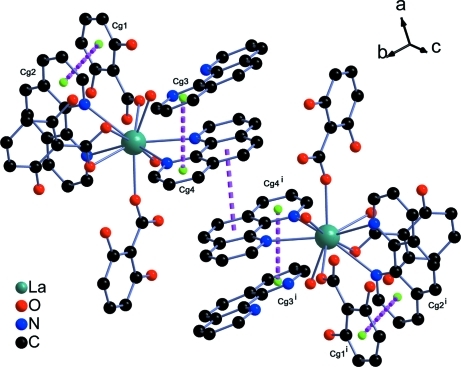
Extensive π···π stacking interaction between DHB and phen, phen and phen. Cg (light green sphere) denotes the centroid of six-membered ring: Cg1 = C39 - C44, Cg2 = N1 / C13 - C16 / C57, Cg3 = N6 / C45 - C49, Cg4 = N3 / C1 - C5. Pink dashed lines show the π - π stacking between ligands [symmetry codes: (i) 1 - x, - y, - z].

## supplementary crystallographic information

### Comment

The chemistry of lanthanide-based metal-organic frameworks is currently of great interest because of their unusual coordination characteristics and optical and magnetic properties (Santos *et al.* 2008). Herein, we report on the preparation and the single-crystal X-ray structure of the novel mononuclear mixed-ligand complex [La(C_7_H_5_O_4_)_3_ (C_12_H_8_N_2_)_2_(H_2_O)_2_][C_12_H_8_N_2_].

The mononuclear structure is shown in Fig. 1. The ten-coordinated geometry of the La^III^ ion is completed by four 2, 6-dihydroxybenzoate (DHB) O atoms, four phenanthroline N atoms and two water O atoms. In one unit cell, there are four complex molecules.The complex forms a ten-coordinated pseudo-bicapped square antiprismatic structure in which the set O6, N3, O14 and N1 and the set N2, O9, N4 and O1 form two approximate squares, respectively. The ninth coordinating atom O5 and tenth coordinating atom O13 are above and under the two planes formed by O6, N3, O14 and N1 and N2, O9, N4 and O1, respectively, and locate at bicapped positions. The O5–La–O13 is 173.32 (7)° close to 180°. Because the coordinating atom O5 is excluded by O6, N3, O14 and N1 (formed above the plane) the bond distance of La^III^–O5 [2.800 (2) Å] is longer than the La^III^–O6 [2.633 (2) Å].

The uncoordinated phen is stabilized by O—H···N hydrogen bonds (Table 1 & Fig. 1) and π - π stacking (Fig. 2) with the coordinated phen [*Cg*3 - *Cg*4 distance = 3.7291 (16) Å, *Cg* denotes the centroid of ring N6 / C45 - C49 for *Cg3*, N3 / C1 - C5 for *Cg4*]. Partial overlap between DHB and phen ligand is observed in the molecular structure, which suggests the existence of intramolecular π-π stacking [Fig. 2, *Cg*1 - *Cg*2 distance = 3.933 (2) Å, *Cg* denotes the centroid of ring C39 - C44 for *Cg1*, N1 / C13 - C16 / C57 for *Cg2*]. Intermolecular π - π stacking is also present in the crystal structure, the face-to-face distance between nearly parallel [dihedral angle = 0.11 (4) °] partial overlapping N3-phen and N3^i^-phen rings is 3.461 (3) Å [symmetry code: (i) 1 - *x*, - *y*, - *z*].

O—H···O hydrogen bonds helping to stabilize the crystal structure are observed in the DHB ligands and between a water molecule and DHB ligands (Table 1 & Fig. 1).

### Experimental

Each reagent was commercially available and of analytical grade. La(NO_3_)_3_^.^6H_2_O (0.136 g, 0.5 mmol), 2, 6-dihydroxybenzoic acid (0.074 g 0.5 mmol), 1,10-phenanthroline (0.090 g, 0.5 mmol) and NaHCO_3_ (0.042 g, 0.5 mmol) were dissolved in a water-ethanol solution (10 ml, 5:5). The solution was refluxed for 4 h, and filtered after cooling to room temperature. Pink single crystals were obtained from the filtrate after 3 d.

### Refinement

The H atoms of water molecules were located in a difference Fourier map and refined with O—H distance restraints of 0.87 (1) Å  for O13 and 0.85 (2) Å for O14. The distances between La and the water H13*a* and H13*b* (H14*a* and H14*b*) atoms were restrained to be equal with an effective stand deviation 0.02. The distances between H13*a* and H13*b* (H14*a* and H14*b*) were further restrained to 1.40 (0.04) [1.39 (0.04)] Å. Other H atoms were positioned geometrically (C—H = 0.93 Å and O—H = 0.82 Å) and refined as riding, with *U*_iso _(H) = 1.2Ueq (C) and *U*_iso_(H) = 1.5Ueq (O). The anisotropic displacement parameters in the direction of some bonds were restrained to be equalinstruction [instruction DELU 0.001 0.001 O1 C38 O2 C38 O4 C44 C38 C39 C41 C42].

### Crystal data

**Table:**

[La(C_7_H_5_O_4_)_3_(C_12_H_8_N_2_)_3_(H_2_O)_2_]·C_12_H_8_N_2_	*F*(000) = 2384
*M**_r_* = 1174.88	*D*_x_ = 1.538 Mg m^−^^3^
Monoclinic, *P*2_1_/*c*	Mo *K*α radiation, λ = 0.71073 Å
Hall symbol: -P 2ybc	Cell parameters from 19311 reflections
*a* = 13.9735 (3) Å	θ = 3.0–29.1°
*b* = 19.6751 (3) Å	µ = 0.92 mm^−^^1^
*c* = 18.6337 (3) Å	*T* = 291 K
β = 98.059 (2)°	Block, pink
*V* = 5072.37 (16) Å^3^	0.50 × 0.46 × 0.42 mm
*Z* = 4	

### Data collection

**Table:**

Oxford Diffraction Gemini S Ultra diffractometer	10312 independent reflections
Radiation source: Enhance (Mo) X-ray Source	7901 reflections with *I* > 2σ(*I*)
graphite	*R*_int_ = 0.022
Detector resolution: 15.9149 pixels mm^-1^	θ_max_ = 26.4°, θ_min_ = 3.0°
ω scans	*h* = −17→17
Absorption correction: multi-scan [ABSPACK in *CrysAlis PRO* (Oxford Diffraction, 2006)]	*k* = −24→22
*T*_min_ = 0.657, *T*_max_ = 0.699	*l* = −23→23
32940 measured reflections	

### Refinement

**Table:**

Refinement on *F*^2^	Primary atom site location: structure-invariant direct methods
Least-squares matrix: full	Secondary atom site location: difference Fourier map
*R*[*F*^2^ > 2σ(*F*^2^)] = 0.031	Hydrogen site location: inferred from neighbouring sites
*wR*(*F*^2^) = 0.086	H atoms treated by a mixture of independent and constrained refinement
*S* = 1.05	*w* = 1/[σ^2^(*F*_o_^2^) + (0.0521*P*)^2^] where *P* = (*F*_o_^2^ + 2*F*_c_^2^)/3
10312 reflections	(Δ/σ)_max_ = 0.001
721 parameters	Δρ_max_ = 0.95 e Å^−^^3^
25 restraints	Δρ_min_ = −0.49 e Å^−^^3^

### Fractional atomic coordinates and isotropic or equivalent isotropic displacement parameters (Å^2^)

**Table:**

	*x*	*y*	*z*	*U*_iso_*/*U*_eq_	
La1	0.370753 (10)	0.081073 (8)	0.238632 (7)	0.03468 (6)	
O9	0.53465 (13)	0.13343 (10)	0.21766 (10)	0.0453 (4)	
O13	0.51050 (13)	0.01072 (11)	0.30096 (9)	0.0464 (5)	
O14	0.21124 (15)	0.03227 (12)	0.17926 (10)	0.0547 (5)	
O1	0.31357 (14)	−0.00908 (10)	0.32214 (10)	0.0490 (5)	
N2	0.43187 (17)	0.13969 (12)	0.37167 (11)	0.0439 (5)	
O11	0.55321 (16)	0.25517 (13)	0.17366 (15)	0.0744 (7)	
H11	0.5250	0.2203	0.1825	0.112*	
N3	0.38115 (16)	0.08988 (11)	0.09173 (11)	0.0406 (5)	
O2	0.42323 (16)	−0.01459 (13)	0.42242 (12)	0.0710 (6)	
N4	0.40066 (16)	−0.03365 (12)	0.15917 (11)	0.0444 (5)	
O5	0.23093 (17)	0.17180 (11)	0.17268 (11)	0.0627 (6)	
O12	0.83139 (16)	0.12522 (14)	0.27052 (17)	0.0824 (8)	
H12	0.7917	0.0943	0.2678	0.124*	
O6	0.36459 (15)	0.21446 (12)	0.22776 (11)	0.0613 (6)	
O10	0.66549 (16)	0.07249 (11)	0.25757 (13)	0.0582 (6)	
O3	0.13794 (19)	−0.04061 (15)	0.33528 (14)	0.0836 (7)	
H3	0.1846	−0.0313	0.3149	0.100*	
O7	0.07660 (16)	0.24216 (15)	0.15335 (17)	0.0860 (8)	
H7	0.1122	0.2097	0.1491	0.129*	
N1	0.23691 (17)	0.12766 (12)	0.32674 (12)	0.0472 (6)	
C5	0.37874 (18)	0.03313 (15)	0.05045 (13)	0.0395 (6)	
C53	0.0631 (3)	−0.0425 (4)	−0.1339 (3)	0.117 (2)	
H53	0.0401	−0.0347	−0.1825	0.140*	
C26	0.2802 (2)	0.34560 (17)	0.23416 (15)	0.0546 (8)	
C57	0.2677 (2)	0.14228 (14)	0.39758 (14)	0.0459 (7)	
O8	0.37449 (19)	0.34162 (15)	0.26058 (17)	0.0866 (8)	
H8	0.3947	0.3036	0.2528	0.130*	
C25	0.2286 (2)	0.28831 (14)	0.20674 (13)	0.0410 (6)	
C32	0.68839 (19)	0.18505 (14)	0.22081 (14)	0.0424 (6)	
C21	0.5021 (3)	0.16812 (17)	0.51614 (17)	0.0680 (10)	
H21	0.5262	0.1768	0.5644	0.082*	
C6	0.38765 (18)	−0.03198 (15)	0.08544 (14)	0.0412 (6)	
C1	0.3779 (2)	0.14897 (16)	0.05841 (14)	0.0482 (7)	
H1	0.3812	0.1882	0.0866	0.058*	
C31	0.62605 (19)	0.12679 (15)	0.23241 (14)	0.0410 (6)	
C39	0.2617 (2)	−0.02689 (15)	0.43750 (16)	0.0533 (6)	
C30	0.1304 (2)	0.29608 (18)	0.18138 (16)	0.0558 (8)	
C20	0.3690 (2)	0.14768 (14)	0.42102 (14)	0.0444 (7)	
N6	0.14051 (19)	0.0521 (2)	0.03302 (15)	0.0701 (9)	
C16	0.2025 (3)	0.15139 (17)	0.44877 (18)	0.0618 (9)	
O4	0.37425 (19)	−0.00989 (16)	0.54696 (13)	0.0834 (8)	
H4	0.4104	−0.0074	0.5160	0.100*	
C24	0.2765 (2)	0.22140 (16)	0.20203 (14)	0.0476 (7)	
C33	0.7897 (2)	0.18107 (17)	0.23974 (18)	0.0575 (8)	
C10	0.3959 (2)	−0.15344 (17)	0.07953 (19)	0.0619 (9)	
H10	0.3937	−0.1938	0.0533	0.074*	
C7	0.3848 (2)	−0.09133 (16)	0.04329 (16)	0.0506 (7)	
C23	0.5250 (2)	0.14743 (16)	0.39463 (15)	0.0538 (8)	
H23	0.5678	0.1424	0.3610	0.065*	
C11	0.4098 (2)	−0.15506 (17)	0.1532 (2)	0.0637 (9)	
H11A	0.4177	−0.1962	0.1779	0.076*	
C4	0.3692 (2)	0.03634 (16)	−0.02681 (14)	0.0457 (7)	
C19	0.4028 (3)	0.16075 (16)	0.49545 (16)	0.0585 (9)	
C3	0.3653 (2)	0.09972 (18)	−0.05871 (15)	0.0575 (8)	
H3A	0.3598	0.1035	−0.1089	0.069*	
N5	0.1552 (2)	−0.0787 (2)	0.0868 (2)	0.0816 (10)	
C27	0.2349 (4)	0.40856 (18)	0.2342 (2)	0.0768 (12)	
H27	0.2689	0.4470	0.2519	0.092*	
C37	0.6493 (2)	0.24582 (17)	0.18986 (16)	0.0537 (7)	
C8	0.3719 (3)	−0.08552 (19)	−0.03447 (18)	0.0632 (9)	
H8A	0.3682	−0.1248	−0.0625	0.076*	
C38	0.3389 (2)	−0.01706 (16)	0.39193 (15)	0.0510 (6)	
C29	0.0853 (3)	0.3571 (2)	0.1822 (2)	0.0738 (10)	
H29	0.0195	0.3610	0.1658	0.089*	
C36	0.7088 (3)	0.29931 (19)	0.1764 (2)	0.0799 (11)	
H36	0.6827	0.3390	0.1547	0.096*	
C22	0.5633 (3)	0.16273 (17)	0.46626 (17)	0.0634 (9)	
H22	0.6294	0.1691	0.4793	0.076*	
C12	0.4120 (2)	−0.09409 (15)	0.19086 (18)	0.0545 (8)	
H12A	0.4221	−0.0957	0.2412	0.065*	
C44	0.2853 (3)	−0.02329 (18)	0.51560 (18)	0.0641 (8)	
C2	0.3696 (2)	0.15638 (18)	−0.01716 (16)	0.0591 (8)	
H2	0.3672	0.1993	−0.0382	0.071*	
C43	0.2142 (3)	−0.0313 (2)	0.55818 (19)	0.0827 (12)	
H43	0.2280	−0.0281	0.6084	0.099*	
C49	0.1184 (2)	0.0010 (3)	−0.01247 (18)	0.0718 (11)	
C18	0.3357 (4)	0.1663 (2)	0.54486 (18)	0.0800 (12)	
H18	0.3580	0.1730	0.5938	0.096*	
C50	0.1264 (2)	−0.0668 (3)	0.0140 (2)	0.0785 (13)	
C9	0.3650 (2)	−0.02496 (19)	−0.06744 (16)	0.0601 (9)	
H9	0.3573	−0.0230	−0.1178	0.072*	
C13	0.1424 (2)	0.12352 (18)	0.3062 (2)	0.0614 (8)	
H13	0.1207	0.1137	0.2579	0.074*	
C40	0.1652 (3)	−0.03815 (18)	0.41086 (19)	0.0700 (10)	
C17	0.2401 (4)	0.1622 (2)	0.5229 (2)	0.0884 (14)	
H17	0.1976	0.1664	0.5568	0.106*	
C34	0.8477 (3)	0.2355 (2)	0.2272 (2)	0.0786 (11)	
H34	0.9143	0.2330	0.2403	0.094*	
C35	0.8069 (3)	0.2928 (2)	0.1955 (3)	0.0868 (12)	
H35	0.8468	0.3287	0.1865	0.104*	
C14	0.0735 (3)	0.13304 (19)	0.3526 (2)	0.0745 (11)	
H14	0.0079	0.1303	0.3354	0.089*	
C45	0.1328 (3)	0.1150 (3)	0.0061 (2)	0.0856 (13)	
H45	0.1488	0.1508	0.0381	0.103*	
C28	0.1376 (4)	0.4122 (2)	0.2073 (2)	0.0817 (13)	
H28	0.1069	0.4542	0.2063	0.098*	
C48	0.0860 (3)	0.0128 (3)	−0.0888 (2)	0.0863 (14)	
C52	0.0727 (4)	−0.1068 (4)	−0.1105 (3)	0.1101 (19)	
H52	0.0600	−0.1425	−0.1432	0.132*	
C15	0.1046 (3)	0.1464 (2)	0.4234 (2)	0.0811 (12)	
H15	0.0597	0.1523	0.4554	0.097*	
C47	0.0801 (3)	0.0789 (3)	−0.1119 (2)	0.1025 (18)	
H47	0.0600	0.0881	−0.1607	0.123*	
C46	0.1027 (3)	0.1308 (3)	−0.0659 (2)	0.1003 (15)	
H46	0.0984	0.1756	−0.0818	0.120*	
C42	0.1231 (4)	−0.0438 (3)	0.5259 (3)	0.1072 (16)	
H42	0.0755	−0.0500	0.5555	0.129*	
C56	0.1608 (3)	−0.1414 (3)	0.1106 (3)	0.1047 (16)	
H56	0.1791	−0.1483	0.1600	0.126*	
C41	0.0965 (3)	−0.0482 (3)	0.4522 (3)	0.1018 (14)	
H41	0.0332	−0.0577	0.4321	0.122*	
C51	0.1028 (3)	−0.1210 (3)	−0.0347 (3)	0.1021 (16)	
C54	0.1127 (4)	−0.1855 (4)	−0.0061 (4)	0.125 (2)	
H54	0.0995	−0.2219	−0.0378	0.151*	
C55	0.1403 (4)	−0.1999 (3)	0.0653 (4)	0.125 (2)	
H55	0.1455	−0.2441	0.0831	0.150*	
H13B	0.508 (2)	−0.016 (2)	0.337 (2)	0.187*	
H13A	0.5699 (11)	0.017 (3)	0.293 (3)	0.187*	
H14B	0.206 (3)	−0.0096 (11)	0.182 (3)	0.187*	
H14A	0.193 (3)	0.047 (2)	0.1363 (16)	0.187*	

### Atomic displacement parameters (Å^2^)

**Table:**

	*U*^11^	*U*^22^	*U*^33^	*U*^12^	*U*^13^	*U*^23^
La1	0.03296 (9)	0.04413 (10)	0.02695 (8)	−0.00167 (7)	0.00423 (6)	−0.00339 (7)
O9	0.0333 (10)	0.0576 (12)	0.0443 (10)	−0.0030 (9)	0.0034 (8)	0.0076 (9)
O13	0.0440 (11)	0.0558 (12)	0.0392 (10)	0.0019 (10)	0.0052 (8)	0.0067 (9)
O14	0.0448 (12)	0.0729 (15)	0.0441 (11)	−0.0047 (11)	−0.0020 (9)	−0.0077 (11)
O1	0.0521 (11)	0.0527 (12)	0.0416 (8)	−0.0008 (10)	0.0042 (7)	0.0015 (9)
N2	0.0494 (14)	0.0491 (14)	0.0330 (11)	0.0024 (11)	0.0049 (10)	−0.0041 (10)
O11	0.0539 (14)	0.0711 (16)	0.0948 (17)	0.0002 (12)	−0.0019 (13)	0.0358 (15)
N3	0.0424 (13)	0.0481 (14)	0.0317 (11)	−0.0012 (10)	0.0067 (9)	−0.0054 (10)
O2	0.0567 (10)	0.0873 (17)	0.0637 (13)	−0.0137 (13)	−0.0094 (9)	0.0200 (13)
N4	0.0443 (13)	0.0493 (14)	0.0388 (12)	0.0021 (11)	0.0028 (10)	−0.0036 (11)
O5	0.0808 (16)	0.0539 (14)	0.0533 (12)	−0.0039 (12)	0.0092 (11)	−0.0055 (11)
O12	0.0392 (13)	0.0785 (18)	0.128 (2)	0.0056 (12)	0.0071 (14)	0.0211 (17)
O6	0.0531 (14)	0.0692 (15)	0.0630 (13)	0.0147 (11)	0.0124 (11)	0.0122 (11)
O10	0.0422 (11)	0.0540 (14)	0.0795 (16)	0.0048 (10)	0.0126 (10)	0.0134 (11)
O3	0.0801 (11)	0.0850 (11)	0.0841 (11)	−0.0047 (9)	0.0056 (8)	−0.0049 (9)
O7	0.0500 (14)	0.094 (2)	0.110 (2)	−0.0117 (14)	−0.0042 (14)	0.0016 (18)
N1	0.0469 (14)	0.0509 (15)	0.0460 (13)	0.0008 (12)	0.0142 (11)	−0.0006 (11)
C5	0.0311 (14)	0.0556 (17)	0.0325 (12)	−0.0028 (12)	0.0067 (11)	−0.0088 (13)
C53	0.081 (3)	0.205 (7)	0.067 (3)	−0.043 (4)	0.019 (2)	−0.040 (4)
C26	0.063 (2)	0.060 (2)	0.0407 (15)	−0.0021 (17)	0.0079 (15)	0.0002 (15)
C57	0.0634 (19)	0.0373 (15)	0.0411 (14)	0.0029 (14)	0.0217 (14)	−0.0012 (12)
O8	0.0741 (18)	0.090 (2)	0.0882 (18)	−0.0214 (14)	−0.0142 (15)	−0.0110 (17)
C25	0.0459 (16)	0.0460 (16)	0.0325 (13)	0.0034 (13)	0.0108 (11)	0.0045 (12)
C32	0.0401 (15)	0.0479 (16)	0.0405 (14)	−0.0038 (13)	0.0102 (12)	−0.0037 (13)
C21	0.103 (3)	0.056 (2)	0.0383 (16)	0.012 (2)	−0.0135 (18)	−0.0127 (15)
C6	0.0326 (14)	0.0520 (17)	0.0389 (13)	0.0009 (12)	0.0048 (11)	−0.0087 (13)
C1	0.0576 (19)	0.0488 (18)	0.0386 (14)	−0.0009 (14)	0.0087 (13)	−0.0023 (13)
C31	0.0392 (16)	0.0506 (17)	0.0346 (13)	−0.0024 (13)	0.0106 (11)	−0.0013 (13)
C39	0.0711 (14)	0.0353 (15)	0.0578 (11)	−0.0019 (15)	0.0236 (10)	0.0032 (14)
C30	0.0511 (19)	0.066 (2)	0.0521 (17)	0.0020 (17)	0.0137 (14)	0.0103 (16)
C20	0.067 (2)	0.0359 (15)	0.0314 (13)	0.0047 (14)	0.0119 (13)	−0.0039 (11)
N6	0.0432 (16)	0.117 (3)	0.0491 (15)	−0.0096 (17)	0.0048 (13)	−0.0182 (19)
C16	0.077 (2)	0.0528 (19)	0.064 (2)	−0.0040 (17)	0.0375 (18)	−0.0104 (16)
O4	0.0902 (17)	0.102 (2)	0.0572 (13)	−0.0043 (17)	0.0078 (11)	0.0098 (15)
C24	0.0527 (18)	0.0587 (19)	0.0332 (13)	0.0005 (15)	0.0125 (13)	0.0038 (14)
C33	0.0423 (17)	0.059 (2)	0.073 (2)	−0.0022 (16)	0.0151 (15)	−0.0038 (17)
C10	0.063 (2)	0.051 (2)	0.071 (2)	0.0026 (16)	0.0066 (17)	−0.0184 (17)
C7	0.0450 (17)	0.057 (2)	0.0504 (16)	−0.0009 (14)	0.0086 (13)	−0.0160 (15)
C23	0.059 (2)	0.059 (2)	0.0416 (15)	0.0050 (16)	0.0020 (14)	−0.0096 (14)
C11	0.067 (2)	0.0455 (19)	0.077 (2)	0.0053 (16)	0.0052 (18)	−0.0001 (17)
C4	0.0418 (16)	0.064 (2)	0.0317 (13)	−0.0021 (14)	0.0074 (12)	−0.0074 (14)
C19	0.095 (3)	0.0451 (18)	0.0349 (15)	0.0059 (17)	0.0076 (16)	−0.0062 (13)
C3	0.062 (2)	0.081 (2)	0.0300 (14)	−0.0061 (17)	0.0077 (14)	−0.0003 (15)
N5	0.0498 (18)	0.102 (3)	0.090 (3)	−0.0112 (18)	0.0001 (17)	−0.018 (2)
C27	0.124 (4)	0.050 (2)	0.059 (2)	−0.013 (2)	0.021 (2)	−0.0079 (16)
C37	0.0485 (17)	0.0566 (19)	0.0572 (17)	−0.0034 (15)	0.0118 (14)	0.0075 (16)
C8	0.067 (2)	0.075 (2)	0.0478 (17)	−0.0051 (18)	0.0102 (16)	−0.0289 (18)
C38	0.0524 (12)	0.0526 (17)	0.0471 (10)	−0.0018 (15)	0.0044 (8)	0.0066 (14)
C29	0.064 (2)	0.079 (3)	0.082 (2)	0.023 (2)	0.0244 (19)	0.021 (2)
C36	0.070 (3)	0.056 (2)	0.117 (3)	−0.0068 (19)	0.024 (2)	0.019 (2)
C22	0.073 (2)	0.059 (2)	0.0507 (18)	0.0082 (17)	−0.0154 (17)	−0.0119 (16)
C12	0.061 (2)	0.050 (2)	0.0510 (17)	0.0053 (15)	0.0017 (15)	0.0000 (14)
C44	0.0784 (17)	0.058 (2)	0.0593 (11)	0.0001 (18)	0.0201 (13)	0.0059 (17)
C2	0.071 (2)	0.065 (2)	0.0427 (16)	−0.0013 (17)	0.0129 (15)	0.0098 (16)
C43	0.098 (3)	0.104 (3)	0.0522 (19)	−0.008 (3)	0.033 (2)	−0.001 (2)
C49	0.0383 (18)	0.127 (4)	0.0511 (19)	−0.016 (2)	0.0109 (15)	−0.019 (2)
C18	0.130 (4)	0.078 (3)	0.0379 (17)	−0.010 (3)	0.031 (2)	−0.0184 (17)
C50	0.0347 (17)	0.130 (4)	0.072 (2)	−0.018 (2)	0.0104 (17)	−0.044 (3)
C9	0.068 (2)	0.078 (2)	0.0352 (14)	−0.0054 (19)	0.0098 (14)	−0.0178 (17)
C13	0.0479 (19)	0.070 (2)	0.070 (2)	−0.0009 (17)	0.0200 (16)	0.0016 (18)
C40	0.080 (3)	0.062 (2)	0.067 (2)	−0.006 (2)	0.006 (2)	−0.0046 (18)
C17	0.128 (4)	0.085 (3)	0.067 (2)	−0.019 (3)	0.063 (3)	−0.025 (2)
C34	0.047 (2)	0.067 (3)	0.124 (3)	−0.0140 (18)	0.022 (2)	−0.009 (2)
C35	0.071 (3)	0.058 (2)	0.137 (4)	−0.022 (2)	0.034 (3)	0.002 (2)
C14	0.050 (2)	0.076 (3)	0.103 (3)	−0.0011 (18)	0.030 (2)	−0.007 (2)
C45	0.061 (2)	0.133 (4)	0.064 (2)	−0.003 (3)	0.0139 (19)	−0.003 (3)
C28	0.112 (4)	0.069 (3)	0.072 (2)	0.039 (3)	0.038 (2)	0.016 (2)
C48	0.051 (2)	0.156 (5)	0.054 (2)	−0.031 (3)	0.0153 (17)	−0.033 (3)
C52	0.081 (3)	0.171 (5)	0.081 (3)	−0.048 (4)	0.020 (3)	−0.066 (4)
C15	0.083 (3)	0.079 (3)	0.094 (3)	−0.001 (2)	0.056 (2)	−0.014 (2)
C47	0.069 (3)	0.182 (6)	0.060 (2)	−0.028 (3)	0.019 (2)	−0.001 (3)
C46	0.071 (3)	0.154 (5)	0.077 (3)	−0.002 (3)	0.015 (2)	0.018 (3)
C42	0.097 (4)	0.133 (4)	0.103 (2)	−0.016 (3)	0.052 (3)	−0.010 (3)
C56	0.062 (3)	0.122 (4)	0.126 (4)	−0.014 (3)	−0.002 (3)	−0.004 (4)
C41	0.079 (3)	0.125 (4)	0.109 (2)	−0.022 (3)	0.040 (3)	−0.017 (3)
C51	0.064 (3)	0.132 (5)	0.114 (4)	−0.032 (3)	0.025 (3)	−0.050 (4)
C54	0.074 (3)	0.134 (6)	0.171 (6)	−0.029 (4)	0.026 (4)	−0.057 (5)
C55	0.077 (3)	0.116 (5)	0.177 (6)	−0.018 (3)	−0.001 (4)	−0.008 (5)

### Geometric parameters (Å, °)

**Table:**

La1—O14	2.534 (2)	O4—C44	1.324 (4)
La1—O13	2.5381 (18)	O4—H4	0.8200
La1—O1	2.5601 (19)	C33—C34	1.383 (5)
La1—O9	2.5904 (18)	C10—C11	1.360 (5)
La1—O6	2.633 (2)	C10—C7	1.394 (4)
La1—N2	2.759 (2)	C10—H10	0.9300
La1—N4	2.763 (2)	C7—C8	1.439 (4)
La1—N3	2.767 (2)	C23—C22	1.400 (4)
La1—O5	2.800 (2)	C23—H23	0.9300
La1—N1	2.810 (2)	C11—C12	1.388 (4)
La1—C24	3.093 (3)	C11—H11A	0.9300
O9—C31	1.275 (3)	C4—C3	1.379 (5)
O13—H13B	0.86 (4)	C4—C9	1.421 (4)
O13—H13A	0.872 (10)	C19—C18	1.408 (5)
O14—H14B	0.829 (19)	C3—C2	1.354 (4)
O14—H14A	0.858 (19)	C3—H3A	0.9300
O1—C38	1.308 (3)	N5—C56	1.308 (6)
N2—C23	1.320 (3)	N5—C50	1.380 (5)
N2—C20	1.367 (3)	C27—C28	1.384 (6)
O11—C37	1.347 (4)	C27—H27	0.9300
O11—H11	0.8200	C37—C36	1.386 (5)
N3—C1	1.316 (3)	C8—C9	1.338 (5)
N3—C5	1.354 (3)	C8—H8A	0.9300
O2—C38	1.235 (3)	C29—C28	1.353 (6)
N4—C12	1.327 (4)	C29—H29	0.9300
N4—C6	1.361 (3)	C36—C35	1.374 (5)
O5—C24	1.249 (3)	C36—H36	0.9300
O12—C33	1.335 (4)	C22—H22	0.9300
O12—H12	0.8200	C12—H12A	0.9300
O6—C24	1.264 (3)	C44—C43	1.365 (5)
O10—C31	1.262 (3)	C2—H2	0.9300
O3—C40	1.407 (4)	C43—C42	1.351 (6)
O3—H3	0.8200	C43—H43	0.9300
O7—C30	1.362 (4)	C49—C50	1.422 (6)
O7—H7	0.8200	C49—C48	1.449 (5)
N1—C13	1.325 (4)	C18—C17	1.343 (6)
N1—C57	1.360 (3)	C18—H18	0.9300
C5—C4	1.428 (3)	C50—C51	1.409 (6)
C5—C6	1.435 (4)	C9—H9	0.9300
C53—C52	1.339 (8)	C13—C14	1.393 (5)
C53—C48	1.385 (7)	C13—H13	0.9300
C53—H53	0.9300	C40—C41	1.327 (5)
C26—O8	1.342 (4)	C17—H17	0.9300
C26—C27	1.392 (5)	C34—C35	1.359 (5)
C26—C25	1.396 (4)	C34—H34	0.9300
C57—C16	1.421 (4)	C35—H35	0.9300
C57—C20	1.425 (4)	C14—C15	1.356 (5)
O8—H8	0.8200	C14—H14	0.9300
C25—C30	1.396 (4)	C45—C46	1.385 (6)
C25—C24	1.485 (4)	C45—H45	0.9300
C32—C37	1.404 (4)	C28—H28	0.9300
C32—C33	1.412 (4)	C48—C47	1.370 (6)
C32—C31	1.474 (4)	C52—C51	1.442 (7)
C21—C22	1.353 (5)	C52—H52	0.9300
C21—C19	1.394 (5)	C15—H15	0.9300
C21—H21	0.9300	C47—C46	1.342 (7)
C6—C7	1.405 (4)	C47—H47	0.9300
C1—C2	1.404 (4)	C46—H46	0.9300
C1—H1	0.9300	C42—C41	1.375 (6)
C39—C40	1.388 (5)	C42—H42	0.9300
C39—C44	1.448 (4)	C56—C55	1.434 (7)
C39—C38	1.476 (4)	C56—H56	0.9300
C30—C29	1.358 (5)	C41—H41	0.9300
C20—C19	1.424 (4)	C51—C54	1.376 (8)
N6—C49	1.323 (5)	C54—C55	1.361 (8)
N6—C45	1.333 (6)	C54—H54	0.9300
C16—C15	1.387 (5)	C55—H55	0.9300
C16—C17	1.423 (5)		
			
O14—La1—O13	124.49 (7)	O6—C24—La1	57.20 (16)
O14—La1—O1	70.88 (6)	C25—C24—La1	163.66 (17)
O13—La1—O1	68.27 (6)	O12—C33—C34	118.7 (3)
O14—La1—O9	145.79 (6)	O12—C33—C32	121.0 (3)
O13—La1—O9	69.28 (6)	C34—C33—C32	120.3 (3)
O1—La1—O9	136.48 (6)	C11—C10—C7	120.0 (3)
O14—La1—O6	109.08 (7)	C11—C10—H10	120.0
O13—La1—O6	126.41 (7)	C7—C10—H10	120.0
O1—La1—O6	136.70 (6)	C10—C7—C6	117.7 (3)
O9—La1—O6	67.17 (6)	C10—C7—C8	123.1 (3)
O14—La1—N2	133.88 (7)	C6—C7—C8	119.1 (3)
O13—La1—N2	72.27 (7)	N2—C23—C22	124.2 (3)
O1—La1—N2	80.05 (6)	N2—C23—H23	117.9
O9—La1—N2	78.49 (6)	C22—C23—H23	117.9
O6—La1—N2	69.82 (7)	C10—C11—C12	118.7 (3)
O14—La1—N4	69.29 (7)	C10—C11—H11A	120.7
O13—La1—N4	68.61 (6)	C12—C11—H11A	120.7
O1—La1—N4	80.98 (7)	C3—C4—C9	122.8 (3)
O9—La1—N4	92.33 (6)	C3—C4—C5	117.8 (3)
O6—La1—N4	141.15 (7)	C9—C4—C5	119.4 (3)
N2—La1—N4	140.53 (7)	C21—C19—C18	122.6 (3)
O14—La1—N3	75.93 (7)	C21—C19—C20	118.0 (3)
O13—La1—N3	110.07 (6)	C18—C19—C20	119.4 (3)
O1—La1—N3	135.14 (6)	C2—C3—C4	120.1 (3)
O9—La1—N3	69.86 (6)	C2—C3—H3A	119.9
O6—La1—N3	82.36 (6)	C4—C3—H3A	119.9
N2—La1—N3	144.11 (7)	C56—N5—C50	119.3 (4)
N4—La1—N3	59.21 (7)	C28—C27—C26	118.1 (4)
O14—La1—O5	62.17 (7)	C28—C27—H27	121.0
O13—La1—O5	173.32 (7)	C26—C27—H27	121.0
O1—La1—O5	116.77 (6)	O11—C37—C36	117.4 (3)
O9—La1—O5	104.92 (6)	O11—C37—C32	121.8 (3)
O6—La1—O5	46.96 (6)	C36—C37—C32	120.8 (3)
N2—La1—O5	103.71 (7)	C9—C8—C7	121.6 (3)
N4—La1—O5	115.73 (6)	C9—C8—H8A	119.2
N3—La1—O5	69.88 (6)	C7—C8—H8A	119.2
O14—La1—N1	76.23 (7)	O2—C38—O1	124.1 (3)
O13—La1—N1	116.29 (6)	O2—C38—C39	117.9 (3)
O1—La1—N1	65.22 (6)	O1—C38—C39	118.0 (3)
O9—La1—N1	129.30 (7)	C28—C29—C30	118.9 (4)
O6—La1—N1	72.69 (7)	C28—C29—H29	120.6
N2—La1—N1	59.08 (7)	C30—C29—H29	120.6
N4—La1—N1	137.96 (7)	C35—C36—C37	118.9 (4)
N3—La1—N1	133.57 (7)	C35—C36—H36	120.5
O5—La1—N1	64.40 (7)	C37—C36—H36	120.5
O14—La1—C24	85.46 (8)	C21—C22—C23	118.5 (3)
O13—La1—C24	149.76 (8)	C21—C22—H22	120.7
O1—La1—C24	126.36 (7)	C23—C22—H22	120.7
O9—La1—C24	88.26 (7)	N4—C12—C11	123.8 (3)
O6—La1—C24	23.79 (7)	N4—C12—H12A	118.1
N2—La1—C24	83.89 (7)	C11—C12—H12A	118.1
N4—La1—C24	134.64 (7)	O4—C44—C43	118.8 (3)
N3—La1—C24	78.80 (7)	O4—C44—C39	121.4 (3)
O5—La1—C24	23.81 (7)	C43—C44—C39	119.8 (3)
N1—La1—C24	62.62 (7)	C3—C2—C1	118.6 (3)
C31—O9—La1	143.79 (17)	C3—C2—H2	120.7
La1—O13—H13B	125 (2)	C1—C2—H2	120.7
La1—O13—H13A	123 (2)	C42—C43—C44	118.6 (4)
H13B—O13—H13A	111 (4)	C42—C43—H43	120.7
La1—O14—H14B	116 (3)	C44—C43—H43	120.7
La1—O14—H14A	114 (3)	N6—C49—C50	119.4 (3)
H14B—O14—H14A	112 (4)	N6—C49—C48	121.3 (5)
C38—O1—La1	128.55 (18)	C50—C49—C48	119.2 (4)
C23—N2—C20	117.8 (2)	C17—C18—C19	121.4 (3)
C23—N2—La1	120.41 (17)	C17—C18—H18	119.3
C20—N2—La1	120.35 (18)	C19—C18—H18	119.3
C37—O11—H11	109.5	N5—C50—C51	120.9 (5)
C1—N3—C5	117.7 (2)	N5—C50—C49	119.9 (4)
C1—N3—La1	121.26 (17)	C51—C50—C49	119.2 (5)
C5—N3—La1	120.66 (17)	C8—C9—C4	121.1 (3)
C12—N4—C6	117.5 (2)	C8—C9—H9	119.5
C12—N4—La1	120.74 (19)	C4—C9—H9	119.5
C6—N4—La1	120.83 (18)	N1—C13—C14	124.0 (3)
C24—O5—La1	91.38 (18)	N1—C13—H13	118.0
C33—O12—H12	109.5	C14—C13—H13	118.0
C24—O6—La1	99.00 (19)	C41—C40—C39	124.2 (4)
C40—O3—H3	109.5	C41—C40—O3	117.3 (4)
C30—O7—H7	109.5	C39—C40—O3	118.5 (3)
C13—N1—C57	117.4 (3)	C18—C17—C16	121.4 (3)
C13—N1—La1	121.97 (19)	C18—C17—H17	119.3
C57—N1—La1	119.32 (18)	C16—C17—H17	119.3
N3—C5—C4	121.8 (3)	C35—C34—C33	119.7 (3)
N3—C5—C6	119.0 (2)	C35—C34—H34	120.1
C4—C5—C6	119.2 (2)	C33—C34—H34	120.1
C52—C53—C48	122.7 (5)	C34—C35—C36	122.2 (3)
C52—C53—H53	118.7	C34—C35—H35	118.9
C48—C53—H53	118.7	C36—C35—H35	118.9
O8—C26—C27	118.4 (3)	C15—C14—C13	118.4 (3)
O8—C26—C25	121.1 (3)	C15—C14—H14	120.8
C27—C26—C25	120.5 (3)	C13—C14—H14	120.8
N1—C57—C16	122.2 (3)	N6—C45—C46	124.8 (5)
N1—C57—C20	118.6 (2)	N6—C45—H45	117.6
C16—C57—C20	119.2 (3)	C46—C45—H45	117.6
C26—O8—H8	109.5	C29—C28—C27	122.6 (4)
C26—C25—C30	117.9 (3)	C29—C28—H28	118.7
C26—C25—C24	121.6 (3)	C27—C28—H28	118.7
C30—C25—C24	120.5 (3)	C47—C48—C53	123.8 (5)
C37—C32—C33	117.9 (3)	C47—C48—C49	117.3 (4)
C37—C32—C31	121.2 (3)	C53—C48—C49	119.0 (5)
C33—C32—C31	120.8 (3)	C53—C52—C51	120.3 (5)
C22—C21—C19	120.2 (3)	C53—C52—H52	119.8
C22—C21—H21	119.9	C51—C52—H52	119.9
C19—C21—H21	119.9	C14—C15—C16	120.7 (3)
N4—C6—C7	122.3 (3)	C14—C15—H15	119.6
N4—C6—C5	118.1 (2)	C16—C15—H15	119.6
C7—C6—C5	119.6 (2)	C46—C47—C48	121.5 (5)
N3—C1—C2	123.9 (3)	C46—C47—H47	119.3
N3—C1—H1	118.1	C48—C47—H47	119.3
C2—C1—H1	118.1	C47—C46—C45	117.5 (5)
O10—C31—O9	122.6 (3)	C47—C46—H46	121.3
O10—C31—C32	118.5 (2)	C45—C46—H46	121.3
O9—C31—C32	118.9 (3)	C43—C42—C41	124.2 (4)
C40—C39—C44	116.1 (3)	C43—C42—H42	117.9
C40—C39—C38	124.5 (3)	C41—C42—H42	117.9
C44—C39—C38	119.3 (3)	N5—C56—C55	124.0 (5)
C29—C30—O7	117.4 (3)	N5—C56—H56	118.0
C29—C30—C25	122.0 (3)	C55—C56—H56	118.0
O7—C30—C25	120.6 (3)	C40—C41—C42	117.0 (4)
N2—C20—C19	121.3 (3)	C40—C41—H41	121.5
N2—C20—C57	119.3 (2)	C42—C41—H41	121.5
C19—C20—C57	119.4 (3)	C54—C51—C50	116.5 (6)
C49—N6—C45	117.7 (4)	C54—C51—C52	123.9 (6)
C15—C16—C57	117.3 (3)	C50—C51—C52	119.6 (6)
C15—C16—C17	123.6 (3)	C55—C54—C51	124.8 (6)
C57—C16—C17	119.1 (3)	C55—C54—H54	117.6
C44—O4—H4	109.5	C51—C54—H54	117.6
O5—C24—O6	119.4 (3)	C54—C55—C56	114.4 (6)
O5—C24—C25	120.8 (3)	C54—C55—H55	122.8
O6—C24—C25	119.8 (3)	C56—C55—H55	122.8
O5—C24—La1	64.81 (17)		

### Hydrogen-bond geometry (Å, °)

**Table:**

*D*—H···*A*	*D*—H	H···*A*	*D*···*A*	*D*—H···*A*
O11—H11···O9	0.82	1.83	2.557 (3)	147
O12—H12···O10	0.82	1.80	2.520 (3)	146
O3—H3···O1	0.82	1.84	2.576 (3)	149
O7—H7···O5	0.82	1.82	2.545 (3)	147
O8—H8···O6	0.82	1.85	2.575 (4)	147
O4—H4···O2	0.82	1.78	2.512 (3)	147
O13—H13A···O10	0.87 (1)	1.92 (2)	2.704 (3)	150 (4)
O13—H13B···O2	0.86 (4)	2.11 (3)	2.762 (3)	132 (4)
O14—H14A···N6	0.86 (2)	1.96 (2)	2.793 (4)	162 (4)
O14—H14B···N5	0.83 (2)	2.26 (4)	2.824 (4)	125 (4)
